# The mast cell exosome-fibroblast connection: A novel pro-fibrotic pathway

**DOI:** 10.3389/fmed.2023.1139397

**Published:** 2023-02-23

**Authors:** Alexandria Savage, Cristobal Risquez, Kazunori Gomi, Ryan Schreiner, Alain C. Borczuk, Stefan Worgall, Randi B. Silver

**Affiliations:** ^1^Silver Laboratory, Department of Physiology and Biophysics, Weill Cornell Medicine, New York, NY, United States; ^2^Division of Pulmonary, Critical Care Medicine, Department of Medicine, Weill Cornell Medicine, New York, NY, United States; ^3^Division of Regenerative Medicine, Department of Medicine, Hartman Institute for Therapeutic Organ Regeneration, Ansary Stem Cell Institute, Weill Cornell Medicine, New York, NY, United States; ^4^Department of Pathology and Laboratory Medicine, New York Presbyterian Hospital-Weill Cornell Medicine, New York, NY, United States; ^5^Department of Pediatrics, Weill Cornell Medicine, New York, NY, United States; ^6^Department of Genetic Medicine, Weill Cornell Medicine, New York, NY, United States; ^7^Drukier Institute for Children’s Health, Weill Cornell Medicine, New York, NY, United States

**Keywords:** mast cells, exosomes, fibroblasts, lung, fibrosis

## Abstract

**Introduction:**

In addition to the traditional activation of resident receptors by release of local mediators, new evidence favors the existence of exosomes in cell-to-cell communication that mediates delivery of specific cargo to modulate recipient cell function. We report that mast cell exosomes are an additional source of pro-fibrotic substances and constitute a unique pathway for the generation of excess collagen.

**Methods:**

We use primary human lung fibroblasts (HLFs) to demonstrate the uptake of labeled exosomes isolated from the human mast cell line HMC-1 (MC-EXOs), previously shown to contain protein cargo in common with human mast cell exosomes.

**Results:**

The MC-EXO uptake by HLF is to the cytosol and increases both proline hydroxylation in HLF lysate and secreted collagen, within 24 h, which is sustained over 72 h, the same time required for transforming growth factor-β (TGF-β) to activate collagen synthesis in the HLFs. Unlike TGF-β, MC-EXO uptake does not induce fibrillar gene activation or invoke the Smad-nuclear transcription pathway. We show that MC-EXO uptake and TGF-β have an additive effect on collagen synthesis in HLF and postulate that MC-EXO uptake by HLFs is a contributing factor to excess collagen synthesis and represents a unique paradigm for understanding fibrosis.

**Discussion:**

It is known that, in the lungs, mast cells are more activated and increase in number with inflammation, injury and viral infection associated with fibrosis. With the reported increased incidence of post-COVID-pulmonary fibrosis (PCPF), data from patients with severe COVID-19 are presented that show an increase in the mast cell number in lung parenchyma, the site of PCPF. Our findings provide a rationale for targeting multiple fibrogenic pathways in the management of lung fibrosis and the use of mast cell exosomes as a biomarker for the prognostic and diagnostic management of evolving fibrotic lung disease.

## Introduction

Traditionally the fibroblast TGF-β-Smad-nuclear transcription axis has been viewed as the dominant system in lung fibrosis in that an increased abundance of TGF-β leads to the subsequent activation of fibrillary genes leading to pathologic collagen production (1, 2). Large-scale clinical studies for idiopathic pulmonary fibrosis (IPF) have focused primarily on TGF-β and associated signaling pathways, however, most of the clinical trial evidence suggests that therapies for IPF are only partially beneficial (3). Several classes of TGF-β blockers have been developed to treat IPF, including inhibitors of latent TGF-β activation, TGF-β blocking antibodies, and receptor kinase inhibitors. Clinically used drugs such as nintedanib which inhibits early events in TGF-β signaling, and pirfenidone which inhibits both production and activity of TGF-β, partially reduce the rate of declining lung function (4, 5) but are not curative, demonstrating the need for other anti-fibrotic therapeutic strategies. Evidence is accruing that favors the relevance of other fibrotic cytokines, chemokines, and growth factors in the development of pulmonary fibrosis (6). Fibroblasts can also be activated by direct contact with leukocytes through adhesion molecules such as intercellular-adhesion molecule 1 (ICAM1) or vascular-cell adhesion molecule 1 (VCAM1), through reactive oxygen species, and complement factor C1 (7).

Mast cells have been implicated in pulmonary fibrosis and reside in close proximity to fibroblasts (8). Mast cell granules may contain large amounts of pro-fibrotic cytokines (e.g., TGF-β, IL-4, IL- 10, and IL-13) and growth factors (e.g., PDGF) (9). Activated mast cells in lung tissue and elevated concentrations of mast cell products (e.g., tryptase, chymase, and histamine) are in bronchoalveolar lavage fluid (BALF) in IPF patients (10, 11). Histamine directly increases fibroblast proliferation *in vitro* and tryptase stimulates the synthesis of type-I collagen by fibroblasts (10, 12). It has been shown that in IPF, mast cell abundance correlates with disease severity (11). Previously, our work has linked mast cells to fibrosis in a murine bleomycin model of lung fibrosis (10). While mast cells are recognized as important modulators of IPF, their role in disease pathogenesis is poorly defined. Also, with the current occurrence of Post-COVID-19 pulmonary fibrosis (PCPF) (13, 14) a more comprehensive understanding of fibrogenesis is essential for identifying early biomarkers and new therapeutics.

Mast cells are recruited at excessive levels with viral infection and are an important component of the cytokine storm response (15) to SARS-CoV-2 (16). It is predicted that up to one-third of SARS-CoV-2 positive patients with persistent respiratory symptoms will show radiologic findings consistent with fibrosis (17). The presence of mast cells in the lung parenchyma of COVID-19 patients with indications of pulmonary fibrosis characterized by fibroblast proliferation, airspace obliteration, and micro-honeycombing, has not yet been documented (14). We present preliminary data showing that mast cells are present in the lung parenchyma of patients who succumbed to COVID-19.

We report that the uptake of mast cell exosomes by human lung fibroblasts (HLFs) and a concomitant increase in collagen production represents a unique fibrogenic pathway. Exosomes, nanovesicles (30–100 nm) released by a variety of cell types, including mast cells, are formed by the inward budding of multivesicular bodies formed from plasma membrane (18). They have been found in BALF, tracheal aspirates, urine, serum, and breast milk (19–24). Exosomes are capable of intercellular communication by transferring their cargo, which can include protein, lipid, mRNAs, and non-coding RNAs to neighboring cells (23, 25, 26). Mast cells can shed exosomes both constitutively and when degranulating (21, 26).

We use exosomes isolated from the human mast cell line HMC-1 (MC-EXO) to demonstrate their uptake by HLFs. We have previously shown, by proteomic analysis, that human lung mast cell exosomes and HMC-1 exosomes express cargos with common proteins that could be fibrogenic (22) but not members of the traditional cytokine and growth factor families. Here we show that the uptake of MC-EXO by HLFs to the cytosol stimulates collagen production and secretion similar to that induced by TGF-β but with different kinetics. Further, the MC-EXO stimulated collagen production utilizes a TGF-β-SMAD independent pathway. We postulate that MC-EXO uptake by HLFs facilitates the downstream post-translational modification of newly synthesized single stranded collagenous peptides thereby enhancing collagen production.

We further demonstrate that lung parenchymal mast cells numbers are increased in COVID-19 patients compared to control transplant lungs. Collectively, our results may inform the use of exosomes with a mast cell signature in BALF or tracheal aspirates as a biomarker for developing pulmonary fibrosis as in IPF and PCPF. Further, transfer of mast cell exosome cargo to neighboring cells like HLFs demonstrates that an alternative pathway with its own set of specific mediators exists that may target the downstream post-translational modification of newly synthesized collagen peptides. Our findings represent a new paradigm for the role of mast cells in fibrosis and identifies a unique mast cell-dependent pathway.

## Materials and methods

### Isolation of human lung fibroblasts from lung tissue specimens

Surgical lung biopsy tissue was obtained from consented patients under protocols approved by the Institutional Review Board of Weill Cornell Medical College and included macroscopically normal surgical waste tissue specimens. Unidentified waste tissue specimens were used for human lung fibroblast cultures. Lung fibroblasts were isolated from human lung tissue specimens. The human lung tissue was digested with type I collagenase solution (Thermo Fisher, Waltham, MA, United States 1%) and then spun down. Cell pellets were resuspended in DMEM/F12 (Corning, Corning, NY, United States) with 10% fetal bovine serum (Hyclone Laboratories, Logan, UT, United States) and 1% penicillin/streptomycin/amphotericin (Corning, Corning, NY, United States) and the cells were plated into T75 tissue culture flasks. Before treatments cells were left quiescent for 24 h. Treatments included exposure to MC-EXO (40 μg total protein), TGF-β (Peprotech, Cranbury, NJ, United States 10 ng/ml) with or without the TGF-βR1 inhibitor SB525334 (Selleck Chemicals, Houston, TX, United States 10 μM). Cells were pretreated with the inhibitor or vehicle [phosphate-buffered saline (PBS) or ethanol] 15 min before treatment with TGF-β and MC-EXO.

### Mast cell exosome isolation

The human mastocytoma cell line, HMC-1.2, was kindly provided by J.H. Butterfield (Mayo Clinic, Rochester, MN, United States). HMC-1.2 cells were cultured in Iscove’s medium containing 10% fetal bovine serum (FBS) (Hyclone Laboratories, Logan, UT, United States), 1% penicillin/streptomycin (Mediatech, Corning, NY, Unites States), and 0.01% 1 thioglycerol (Sigma Aldrich, St. Louis, MO, United States). Cells were grown to 1 × 10^6^ cells/ml in complete medium and maintained at 37°C and 5% CO_2_. Isolation and characterization of HMC-1 supernatant exosomes were done as routinely performed in our laboratory (22). Briefly, HMC-1 exosomes were collected from HMC-1 cells maintained for 24 h. HMC-1 supernatants from 24 h cultures were pelleted by centrifugation at 500 *g* for 10 min. The supernatant was centrifuged at 12,000 *g* for 20 min. Exosomes were then harvested by centrifugation at 100,000 *g* for 70 min. The exosome pellet was resuspended in 20 ml of PBS and collected by ultracentrifugation at 100,000 *g* for 70 min (Beckman Ti70, Brea, CA, United States). Exosome size and particle number were analyzed by using the DS500 nanoparticle characterization system (NanoSight, Salisbury, United Kingdom) equipped with a blue laser (405 nm).

To visualize MC-EXO uptake by HLF, exosomes were fluorescently labeled using either PKH67 (green fluorochrome, Sigma Aldrich, St. Louis, MO, United States 0.02 μl/μg total protein) or CellVue Claret (far red fluorochrome, Sigma Aldrich, 0.02 μl/μg total protein). Labeled exosomes were washed in 35 ml of PBS, collected by ultracentrifugation as described above and resuspended in PBS. HLF plated on coverslips were exposed for 60 min to fluorescent PKH-67 (fluorescein) labeled MC-EXOs and then washed with PBS and viewed under transmitted light with either a 10X or 60X objective and an inverted epifluorescence microscope (Nikon Eclipse TE 2000-U, Melville, NY, United States) interfaced to a SPOT Insight 2 megapixel color camera (Diagnostic Instruments, Sterling Heights, MI, United States). For visualization on a confocal microscope, after HLF underwent 24 h of transient transfection using Lipofectamine 2000 (Thermo Fisher, Waltham, MA, United States) with mNeonGreen-KDEL, a fluorescent marker of the endoplasmic reticulum, HLF were exposed to the CellVue Claret labeled MC-EXO for 60 min and then imaged on a Zeiss Cell Observer SD confocal microscope with a Yokogawa CSU-X1 spinning disk, Plan-Apochromat × 63/1.4 M27 objective paired with a × 1.2 adapter to a Photometrics Evolve 512 electron multiplying charge coupled device (EMCCD) camera.

### Hydroxyproline assay

Hydroxyproline was measured in HLF lysates as directed (Sigma Aldrich, St. Louis, MO, United States). Briefly, cells were dried and homogenized then hydrolyzed in order to break down proteins. Chloramine T was added and incubated in the wells followed by addition of dimethylaminobenzaldehyde (DMAB) reagent. Standards were processed in the same way. Samples and standards were read in a Bio-Rad iMark plate reader at 550 nm.

### Soluble collagen assay (Sircol)

Soluble collagen was measured in HLF supernatants *via* Sircol assay (Biocolor, Newtownabbey, United Kingdom) performed as directed. Briefly, Sircol dye reagent was added to 700 μl of supernatant and incubated with shaking for 30 min in order to form a collagen-dye complex. The complex was pelleted and washed in an acid-salt wash to remove unbound dye. Finally, alkali reagent was added to dissolve the dye. Samples were read at 550 nm and normalized to standards run at the same time with collagen expressed in units of μg/700 μl. In some experiments cell lysates were measured to quantify total protein/well for normalization using the Bradford Protein Assay (Bio-Rad, Hercules, CA, United States) where collagen is expressed as μg/μg total protein. Changes in collagen secretion are compared to that measured in unstimulated HLFs.

### Western blots

Aliquots of HLF incubated with TGF-β or MC-EXO were homogenized and the protein amounts determined by the Bio-Rad DC protein assay (Bio-Rad, Hercules, CA, United States). Primary antibodies used were rabbit polyclonal Smad2/3 (Cell Signaling, Danvers, MA, United States 8685, 1:1000 dilution) rabbit polyclonal phosphorylated Smad2/3 (Cell Signaling, Danvers, MA, United States 8828, 1:1000 dilution), and rabbit polyclonal GAPDH (Abcam, Waltham, MA, United States ab9485, 1:1000 dilution). GAPDH was used as a loading control. Western blot quantification was performed with ImageJ (NIH, Bethesda, MD, United States).

### RT-PCR

For RNA analysis of HLF lysates, cell pellets were dissolved in RNeasy lysis buffer (Qiagen, Germantown, MD, United States). RNA was isolated and quantified as above, and 1 μg of RNA was reverse transcribed by use of the High Capacity RNA-cDNA kit (Applied Biosystems, Waltham, MA, United States). qPCR was performed at 1/4 dilution with fast SYBR Green Master Mix (Applied Biosystems, Waltham, MA, United States) for human COLI (collagen I) and COLIII (collagen III) genes. GAPDH was used as the housekeeping gene and expression was constant. qPCR was done on a Step One real- time PCR system (Applied Biosystems, Waltham, MA, United States). Human primer sequences are listed in Table 1.

**TABLE 1:** Human qPCR primers.

COLI forward	CCTGTCTGCTTCCTGTAAACTC
COLI reverse	GTTCAGTTTGGGTTGCTTGTC
COLIII forward	CCTCATTAGTCCTGATGGTTCTC
COLIII reverse	GGTTAGGGTCAACCCAGTATTC
GAPDH forward	CAACAGCACAGGAGAG
GAPDH reverse	CTACATGGCAACTGTGAGGAG

### Human lung tissue

COVID-19 and control lung samples were collected at Weill Cornell-New York Presbyterian. All COVID-19 patients tested positive for SARS-CoV-2 by nucleic acid testing of nasopharyngeal swabbed secretion and the COVID-19 lung samples were also tested for SARS-CoV-2 positivity as previously described (27). EM was performed in lung tissues using a transmission electron microscope (TEM, HT-7800). Immunohistochemistry was carried out to identify SARS-CoV-2 viral spike protein (Genetex clone 1A9 at 1:75 dilution with 20-min antigen retrieval at pH 9.0 on Leica Bond III automated instrument). SARS-CoV-2 RNA was detected in lung by RNAscope technology (Advanced Cell Diagnostics, Newark, CA, United States), using SARS-CoV-2 2019-S (cat. 848561), for detection of viral spike protein-encoding RNA. RNA integrity was assessed using a probe targeting the Ubiquitin C housekeeping gene. Positives were confirmed using RNA *in situ* for spike protein encoding RNA. Autopsies were performed at New York Presbyterian-Weill Cornell Medicine, New York City, and conducted in accordance with the principles of the Declaration of Helsinki and approved by the Medical Ethical Committee of New York Presbyterian-Weill Cornell Medicine. Written informed consent for autopsy study was obtained from next of kin and this was the sole selection criterion. According to the New Coronavirus Pneumonia Prevention and Control Program (7th edition), the diagnosis of COVID-19 was confirmed by radiologic features of viral pneumonia, and clinical symptoms. 10 COVID-19 samples were collected and four Control samples are anonymized lung tissue derived from excess donor lung tissue not used for transplantation. Clinical characteristics of patients are summarized in Table 2.

**TABLE 2:** Main clinical data of patients.

Patients	SARS-CoV-2 in lung	Age	Gender	Comorbidities	Symptoms
Control 1	N/A	Unknown	Unknown	Unknown	Unknown
Control 2	N/A	Unknown	Unknown	Unknown	Unknown
Control 3	N/A	Unknown	Unknown	Unknown	Unknown
Control 4	N/A	Unknown	Unknown	Unknown	Unknown
COVID 1	Negative	46	M	DM2, HTN, and asthma	Fever and SOB
COVID 2	Positive	56	F	DM2, HTN, and sickle cell	Fever, cough, and SOB
COVID 3	Positive	64	M	DM2, ESRD, and HTN	Cough and SOB
COVID 4	Negative	87	F	DM2, CAD, and dementia	Fever and SOB
COVID 5	Negative	70	F	None	SOB
COVID 6	Positive	30	F	Pulmonary valve atresia and diabetes	SOB
COVID 7	Negative	69	M	HTN, hyperlipidemia, obesity, and diabetes	Fever, cough, and SOB
COVID 8	Negative	71	F	AML and HTN	Fever, cough, and SOB
COVID 9	Positive	87	M	HTN, DM2, and dementia	Fever, cough, and SOB
COVID 10	Positive (low)	72	F	COPD and uterine cancer	SOB

DM2, diabetes mellitus, Type 2; HTN, hypertension; ESRD, end-stage renal disease; CAD, coronary artery disease; AML, acute myeloid leukemia; COPD, chronic obstructive pulmonary disease; SOB, shortness of breath.

### Lung tissue sampling, processing, and analysis

Lung tissue was obtained from autopsies with a postmortem interval ranging from 1 to 4 days during March 19 to April 25, 2020 as previously described (27). Briefly, a median of 19 (from 8 to 30) lung tissue blocks including subpleural and central parts, were sampled, along with transverse tracheal and bronchus sections. Tissue specimens were fixed in 10% formalin. Immunohistochemistry was carried out to identify SARS-CoV-2 viral spike protein (Genetex clone 1A9 at 1:75 dilution with 20-min antigen retrieval at pH 9.0 on Leica Bond III automated instrument). SARS-CoV-2 RNA was detected in trachea and lung by RNAscope^®^ technology (Advanced Cell Diagnostics, Newark, CA, United States), using SARS-CoV-2 2019-S (cat. 848561), for detection of viral spike protein-encoding RNA. RNA integrity was assessed using a probe targeting the UBC (Ubiquitin C) housekeeping gene. All positives for spike protein IHC were confirmed using RNA *in situ* for spike protein-encoding RNA.

### Tissue histochemical techniques

Fixed lung samples were stained for mast cells which were quantified and visually differentiated to the medium sized airways (0.5–5.0 mm) and parenchyma, and normalized to total tissue area. Before beginning any staining, slides with 7 μm sections were deparaffinized, rehydrated, and washed in distilled water. Slides were stained with hematoxylin and eosin in order to visualize lung structure. Mast cells were detected using both the glycoprotein avidin (conjugated to HRP, Vector Laboratories, Burlingame, CA, United States) (28, 29) and toluidine blue, which is the classic metachromatic stain for mast cells (30, 31). Conjugated avidin is a well-defined histochemical method for identifying human mast cells, which express heparin proteoglycans in the secretory granules (32), binding specifically to the proteoglycan granules (28, 29). Deparaffinized sections were exposed to avidin-HRP (1:500 dilution) and then NovaRED (Vector Laboratories, Burlingame, CA, United States) was used as the chromogen substrate. Each lung section was analyzed for mast cells and quantified as cells per square millimeter of lung tissue. For all histological analyses, de-identified tissue sections were viewed under transmitted light with either a 10X or 60X objective and an inverted epifluorescence microscope (Nikon Eclipse TE 2000-U, Melville, NY, United States) interfaced to a SPOT Insight 2 megapixel color camera (Diagnostic Instruments, Sterling Heights, MI, United States). Whole slide image specimens were scanned and stored on the imaging work station as routinely performed in the lab. When quantifying cell number and determining areas of tissue, Metamorph software (version 6.2; Universal Imaging, West Chester, PA, United States) was used. All histological analyses and cell number quantifications were performed by two investigators in a blinded fashion.

### Statistics

Statistical comparisons were conducted by one-way ANOVA or unpaired two-tailed Student’s *t*-test, whereas a paired *t*-test was used to compare values within the same group with GraphPad Prism 5.0 software (San Diego, CA, United States). A 95% confidence level was considered statistically significant. Values are expressed as means ± SEM.

## Results

**FIGURE 1:**
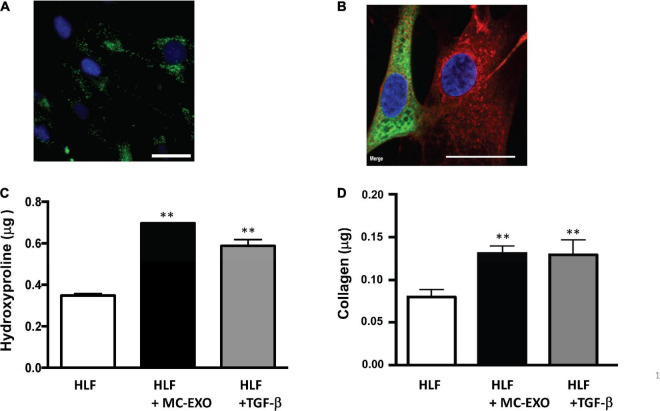
MC-EXO are taken up by HLFs and stimulate collagen synthesis. **(A)** Representative epifluorescence image of HLF uptake of MC-EXO labeled with PKH-67 (green). Nuclei are labeled with 4′,6-diamidino-2-phenylindole (DAPI) (blue). Scale bar = 15 μm. **(B)** Representative confocal image of HLF uptake of MC-EXO labeled with CellVue Claret Far Red (red). HLFs were transiently transfected with mNeonGreen-KDEL, a fluorescent marker of the endoplasmic reticulum. Nuclei are labeled with DAPI (blue). Note that only the HLF on the left was successfully transfected with the mNeonGreen-KDEL marker. Scale bar = 15 μm. **(C)** Hydroxyproline content of lysates from HLF, HLF incubated with MC-EXO (40 μg total protein) or TGF-β (10 ng/ml) at the 72 h time point. All assays performed in triplicate and normalized to total protein (μg). ^**^P < 0.01 versus HLF, *n* = 5 samples/group. **(D)** Secreted collagen in supernatants of HLF, HLF incubated with MC-EXO (40 μg total protein) or TGF-β (10 ng/ml) for 72 h. All assays were performed in triplicate and normalized to total protein (μg). ^**^P < 0.01 versus HLF, *n* = 5 samples/group.

To establish whether MC-EXO can be taken up by HLFs, we incubated HLFs with labeled MC-EXOs (green) and visualized the HLFs under epifluorescence light (Figure 1A). The MC-EXO uptake appeared to be confined to the HLF cytosol in that labeled exosomes were excluded from the nucleus (blue). This MC-EXO localization in HLFs was further confirmed by confocal microscopy as seen in the representative image showing that labeled MC-EXOs (red) are limited to cytosol and endoplasmic reticulum (green) and not observed in the nucleus (blue) (Figure 1B).

To ascertain whether the MC-EXO that are internalized by HLFs can modulate cell function, we compared the proline hydroxylation and the secreted collagen contents from HLFs exposed for 72 h to MC-EXOs or TGF-β, which is the time it takes for TGF-β to activate fibrillar genes and synthesize collagen. MC-EXO significantly increased the proline hydroxylation content of HLF homogenates (Figure 1C) and secreted collagen (Figure 1D) by HLFS and of the same magnitude as that measured with TGF-β stimulation of HLFs.

**FIGURE 2:**
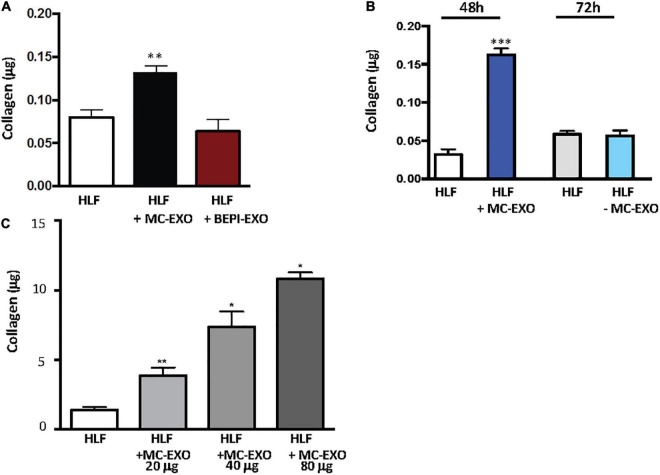
Stimulation of collagen production by MC-EXO uptake in HLFs. **(A)** Secreted collagen was measured in the supernatants of HLF, HLF + MC-EXO (40 μg total protein), HLF + TGF-β (10 ng/ml), and HLF + bronchial epithelial cell exosomes (BEPI)-EXO (40 μg total protein) after 72 h. All assays performed in triplicate and normalized to total protein (μg). ^**^P < 0.01 versus HLF, *n* = 5 samples/group. **(B)** Secreted collagen measured in the supernatants of HLF and HLF + MC-EXO (40 μg total protein) at the 48 h time point and then at the 72 h time point where the MC-EXO have been removed from the supernatant after 48 h. All assays performed in triplicate and normalized to total protein and normalized to total protein (μg). ^***^P < 0.001 versus HLF, *n* = 3 samples/group. **(C)** Secreted collagen measured in the supernatants of HLF and HLF with varying doses of MC-EXO. All assays performed in triplicate and normalized to total volume (700 μl). *P < 0.05, ^**^P < 0.01. *n* = 3 samples/group.

To see if other lung cell exosomes behave similarly to MC-EXO we incubated HLFs with lung bronchial epithelial cell exosomes (BEPI-EXOs), known to be taken up by lung airway cells and associated with COPD (33), and measured the collagen secretion (Figure 2A). Uptake of BEPI-EXOs does not lead to secretion of collagen by HLF showing that MC-EXO uptake and stimulation of collagen synthesis in HLF is not a generalized phenomenon and is dependent on exosome-specific cargo. Additionally, it was determined that MC-EXO uptake by HLF and the concomitant secretion of collagen is dependent on the presence and amount of MC-EXO in the incubation media (Figures 2B, C). Figure 2B shows that as long as MC-EXOs are present in the media bathing HLF they can be taken up and stimulate collagen production. If, after 48 h, as in Figure 2B, MC-EXO are then removed from the bathing media, the stimulatory effect of MC-EXO disappears as seen at the 72 h time point, where the HLF collagen secretion is not different from the control.

The effect of MC-EXO on HLF collagen secretion is also dose-dependent (Figure 2C.) The more MC-EXO are available for uptake in the media the greater the collagen production by the HLF; a doubling of the amount of MC-EXO available for uptake yields a significant increase in collagen secretion. These results suggest that there is a direct effect of the amount of MC-EXO available for uptake by HLF with abundance of secreted collagen.

**FIGURE 3:**
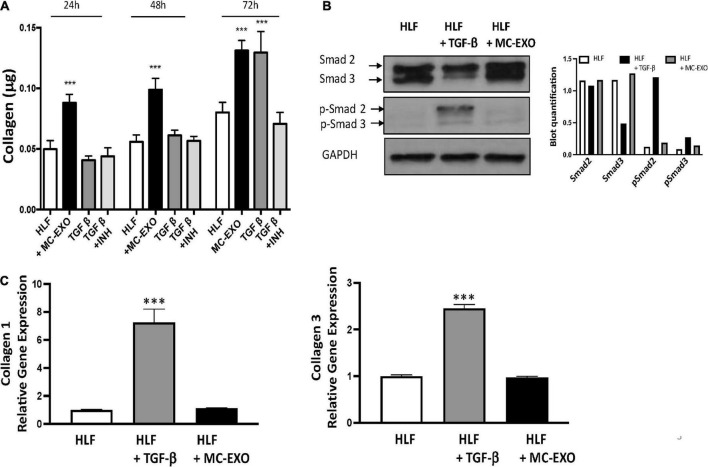
Uptake of MC-EXO by HLF stimulates collagen synthesis *via* a Smad-independent pathway and with different kinetics from TGF-β. **(A)** MC-EXO (40 μg total protein, black bars) and TGF-β (10 ng/ml, dark gray bars) stimulated collagen secretion in HLFs after 24, 48, and 72 h and in untreated HLFs. Shown is the inhibition of TGF-β stimulated collagen secretion in HLFs by the TGF-βR1 inhibitor SB525334 (TGF-βR1 INH, 10 μM, light gray bars). All assays performed in triplicate and normalized to total protein (μg). ^***^P < 0.001 versus HLF, *n* = 6 samples/group. **(B)** Western blot of HLF lysates at 24 h and after treatment with TGF-β (10 ng/ml) or MC-EXO (40 μg total protein). Smad 2/3 and p-Smad 2/3 show signaling *via* the TGF-β pathway. GAPDH is loading control. Western blot was performed twice with associated quantification. **(C)** Collagen 1 and collagen 3 mRNA expression in HLFs and HLFs treated with either TGF-β or MC-EXO analyzed by RealTime qPCR and normalized to GAPDH mRNA. ^***^P < 0.001, *n* = 4 samples/group.

We next compared the time courses of MC-EXOs and TGF-β stimulation on collagen production in HLFs (Figure 3A). MC-EXO uptake by HLFs and the concomitant increase in collagen secretion was measurably significant within the first 24 h of exposure to MC-EXO in the bathing media and continued to increase over the next 2 days (black bars). In contrast, stimulation of HLF by TGF-β shows a time course that is distinct from MC-EXO. HLF were incubated with TGF-β, with and without the TGF-βR1 inhibitor, SB525334. Figure 3A shows that 72 h are necessary to detect measurable collagen secretion in TGF-β stimulated HLF (gray bars) and demonstrates that MC-EXOs and TGF-β induce collagen production with different kinetics.

Inasmuch as MC-EXO uptake by HLF stimulates collagen synthesis by modulating the host cell function with different kinetics from that of TGF-β, we analyzed whether MC-EXOs act *via* the canonical TGF-β-Smad signaling pathway to stimulate HLF collagen production. Western blots of HLF homogenates incubated with MC-EXO or TGF-β for 72 h were probed for phosphorylated Smad. As shown in Figure 3B, phosphorylated Smad was only expressed in HLF homogenates incubated with TGF-β. HLF exposed to MC-EXO do not express phosphorylated Smad demonstrating that the Smad pathway is not activated by MC-EXO taken up by HLF. Additionally, MC-EXO uptake by HLF does not lead to upregulation of the expression of fibrillar genes collagen 1 and collagen 3 unlike that observed with TGF-β (Figure 3C). However, MC-EXO as well as TGF-β leads to increased HLF production of hydroxyproline content and collagen secretion (Figure 1). These results suggest that HLF uptake of exosomes stimulates collagen synthesis downstream from the TGF-β–Smad-nuclear transcription axis and may represent a novel Smad-independent pathway. Despite these findings, a deeper analysis, that is beyond the scope of the present study, is necessary to determine whether the canonical Smad family of proteins plays a role in the stimulation of collagen synthesis by MC-EXO uptake by HLFs.

**FIGURE 4:**
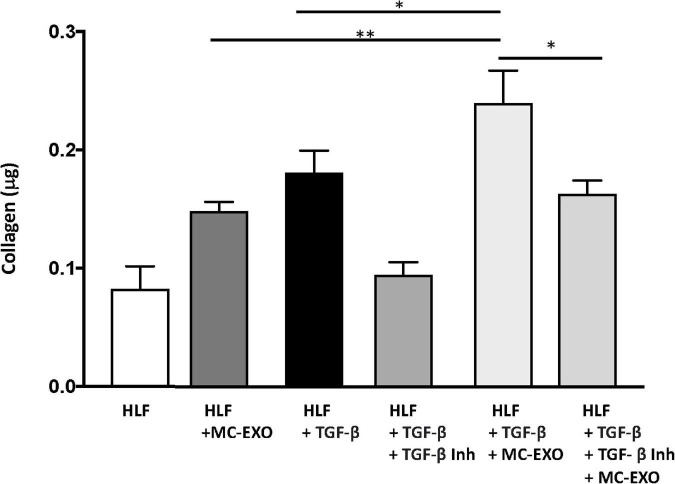
MC-EXOs and TGF-β have an additive effect on collagen synthesis in HLFs. Secreted collagen measurements in the supernatants of HLF s and HLFs incubated with MC-EXO (40 μg) or TGF-β (10 ng/ml) with and without the TGF-βR1 inhibitor SB525334 (10 μM) for 72 h. All assays were performed in triplicate and normalized to total protein (μg). *P < 0.05, ^**^P < 0.01, *n* = 5 samples/group.

To further confirm this finding we measured collagen secretion at the 72 h time point, in HLF incubated with MC-EXO and/or stimulated with TGF-β with and without the TGF-βR1 inhibitor, SB525334. Both MC-EXO and TGF-β independently stimulated collagen synthesis (Figure 4). When MC-EXO and TGF-β were added simultaneously to the media bathing the HLF, the amount of collagen synthesized was greater than each component. When the TGF-βR1 inhibitor was included in the MC-EXO and TGF-β media, the amount of collagen produced reflected that produced by the MC-EXO. Thus, MC-EXO and TGF-β have an additive effect on collagen synthesis in HLF and show that two independent processes exist to modulate collagen synthesis in HLF.

These results show that mast cells play an even greater role in lung fibrosis than previously understood and may have prognostic and diagnostic importance in disease progression. Due to the high prevalence of respiratory failure in patients with severe manifestations of COVID-19, including acute respiratory distress syndrome, vascular leakiness, and marked fibrotic lung parenchymal remodeling, the probability of developing PCPF fibrosis is high (13, 14). We therefore assessed whether lungs from COVID-19 patients had more mast cells in areas prone to developing fibrosis, compared to control lung samples.

**FIGURE 5:**
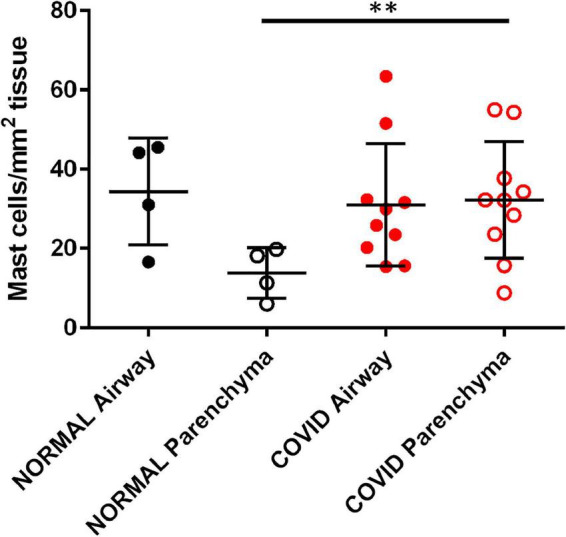
SARS-Cov-2 infection increases lung parenchymal mast cells. Cadaveric lungs from patients infected with SARS-CoV-2 (*n* = 10) or biopsies from donor lungs tissue prior to transplant (*n* = 4) were stained with the mast cell marker avidin HRP. Mast cells were quantified in parenchyma and airway walls (medium sized airways, 0.5–5.0 mm) ^**^P < 0.01 SARS-Cov-2 parenchyma versus normal parenchyma.

Fixed lung samples were stained for mast cells which were quantified and visually differentiated to the airways (medium sized airways, 0.5–5.0 mm) and parenchyma, and normalized to total tissue area (27). The numbers of mast cells residing in airways were similar in controls and COVID-19 samples, reflecting their normal tissue distribution. However, mast cell numbers were significantly increased in the parenchyma of COVID-19 lung samples compared to control samples (Figure 5). Overall, there is a clear increase in the number of mast cells in lung parenchyma of the COVID-19 patient lung samples. Though not a large sample size the implications of this observation may relate to the role of mast cells in PCPF. Mast cells could be a new drug target for abating the long-term effects of COVID-19 infection.

## Discussion

The traditional TGF-β–Smad-nuclear transcription axis plays a critical role in the regulation of extracellular matrix gene expression (34). Overexpression of TGF-β is believed to contribute to the development of tissue fibrosis and activation of Smad proteins; TGF-β receptor kinase substrates that translocate into the cell nucleus to act as transcription factors that activate fibrillar genes and collagen biosynthesis. Here, we report that MC-EXO are acting through an alternate downstream pathway, and that this mast cell–fibroblast intercellular communication constitutes a unique means for generating collagen that contributes to fibrosis. Our findings that labeled MC-EXO taken up by HLF appear to only localize to the cytosol and not nuclei (Figure 1), consistent with published results that visualize uptake of exosomes by lung fibroblasts that track to the endoplasmic reticulum (35), and do not stimulate fibrillar genes (Figure 3), further suggest a downstream and post-translational effect of MC-EXO on the modification of newly synthesized collagenous peptides in the HLF. Additionally, based on our data showing that the inhibition of the TGF-β pathway with the TGF-βR1 inhibitor, SB525334 does not block MC-EXO collagen production (Figure 4), we conclude that the MC-EXO uptake by HLF is an independent pathway that can modulate collagen production.

The *in vitro* experiments in this study focus on MC-EXO and their fibrotic potential, though we have not yet identified the specific MC-EXO cargo responsible. We previously demonstrated *via* proteomic and biochemical analyses that human lung mast cell exosomes and MC-exosomes have very similar proteomic profiles but do not express the classic fibrogenic cytokines and growth factors like TGF-β or PDGF, respectively (36). Further experiments need to be done to investigate the mast cell exosome cargo acting downstream, post-translationally, and responsible for the increase in proline hydroxylation and collagen production with exosome uptake by HLFs.

We are left to consider the physiological significance of MC-EXO uptake by HLF and the concomitant functional modulation of collagen synthesis. When mast cells and other immune cells infiltrate the lung parenchyma in response to injury, the inflammation initiates a cascade of events triggering fibrosis (15). This includes release of cytokines like TGF-β and other profibrotic mediators in close proximity to fibroblasts leading to activation of the TGF-β-Smad-nuclear transcription pathway. The findings presented in the present *in vitro* study advance our understanding of fibrosis in that we identify an additional pathway in the HLFs with the capacity to increase collagen production *via* post-translational modification of newly synthesized collagenous peptides. This pathway is downstream of the TGF-β-Smad-nuclear transcription pathway.

**FIGURE 6:**
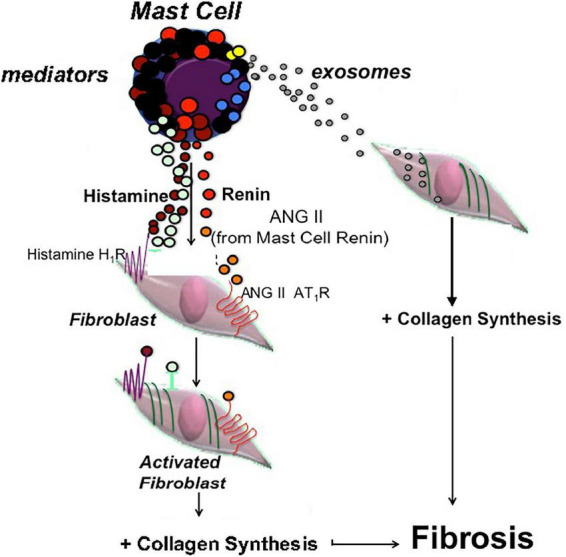
Cartoon depicting two parallel pathways in mast cells leading to fibroblast activation and excess collagen production. One pathway depicts classic mast cell degranulation with release of pro-fibrotic mediators like histamine and renin (ANG II), acting on the resident receptors on fibroblasts that activate the collagen synthesis pathway. The other pathway is the newly identified exosome pathway where shed exosomes are taken up in the cytosol of fibroblasts leading to proline hydroxylation and increased collagen production.

One may ask if this MC-EXO-HLF pathway is relevant compared to the receptor-mediated canonical pathway. Under normal conditions, where mast cells in the lung parenchyma are few in number. However, with lung injury and viral infection, as in IPF and COVID-19 infection (11, 15), respectively, mast cell numbers increase, and they may release fibrotic mediators and exosomes near neighboring fibroblasts to exacerbate collagen production. *In vivo*, mast cells are in close proximity to fibroblasts. With mast cell degranulation and the concomitant release of mediators, like histamine and renin (angiotensin), for example, resident receptors on neighboring fibroblasts can be activated (10) leading to fibrillar gene activation, nuclear transcription, and collagen synthesis. In one of our previous studies we stained fixed sections of lung tissue from IPF patients for mast cells and fibroblasts and found an abundance of both in close proximity (10). Here we report a novel pro-fibrotic pathway where shed mast cell exosomes are taken up in the cytoplasm of fibroblasts and facilitate proline hydroxylation and increased collagen secretion. A cartoon depicting both mast cell pathways is depicted in Figure 6.

In lung injury and viral infection, the increased presence of mast cells in lung parenchyma may yield greater release of mediators and exosomes and serve to promote excess collagen production. In these cases, the presence of exosomes with a mast cell signature in BAL fluid or tracheal aspirates may represent early indicators of potentially progressive disease. Although the clinical data from cadaveric lungs were obtained on a small cohort of patients, the increased population of mast cells in the parenchyma is a potential marker for those patients who will experience a more severe respiratory post-COVID course. Early intervention in the prevention and/or attenuation of IPF and PCPF is not yet possible. Identification of biomarkers for IPF and PCPF will greatly enhance efforts in this area. Our results from this and our previous studies (10, 22) suggest that mast cell products like exosomes may be viable candidates with prognostic and diagnostic value. Further studies on patient-derived mast cell exosomes will be needed to definitively proof their use as a biomarker for developing fibrosis. Exosomes have been isolated from BAL fluid of patients with sarcoid (37) asthma (24), and chronic obstructive pulmonary disease (33). We have previously demonstrated that exosomes isolated from the tracheal aspirates of patients with chronic lung disease of prematurity have a mast cell signature (22). Our current data show that mast cell exosomes are capable of transferring cargo that modifies fibroblast function and could contribute to tissue remodeling and destruction associated with IPF and PCPF. Future work will need to analyze fibroblasts and their possible intercellular contacts to mast cells in COVID-19-infected lung tissue. Our findings open the door for future studies on mast cell exosomes as a novel means of transferring mast cell cargo to neighboring cells. Establishing the mechanisms responsible for damage in the injured or viral infected lung will provide valuable insights for advancing new and effective therapies in the treatment of IPF and PCPF.

## Data availability statement

The original contributions presented in this study are included in the article/supplementary material, further inquiries can be directed to the corresponding author.

## Ethics statement

The studies involving human participants were reviewed and approved by the Medical Ethical Committee of New York Presbyterian-Weill Cornell Medicine. The patients/participants provided their written informed consent to participate in this study.

## Author contributions

AS, CR, KG, and RS performed the experiments and analyzed the data. AS, CR, and RBS interpreted the results of experiments. AS, CR, AB, SW, and RBS contributed to the conception and design of the study. AS, CR, RS, and RBS prepared the figures. AS, SW, AB, and RBS drafted the manuscript. All authors contributed to manuscript revision and approved the final version of the manuscript.
